# Comparative Ubiquitome Analysis under Heat Stress Reveals Diverse Functions of Ubiquitination in *Saccharina japonica*

**DOI:** 10.3390/ijms21218210

**Published:** 2020-11-03

**Authors:** Zhang Pengyan, Liu Fuli, Chen Siqing, Liang Zhourui, Wang Wenjun, Sun Xiutao

**Affiliations:** 1 Key Laboratory of Sustainable Development of Marine Fisheries, Ministry of Agriculture and Rural Affairs, Yellow Sea Fisheries Research Institute, Chinese Academy of Fishery Sciences, Qingdao 266071, China; zhangpy@ysfri.ac.cn (Z.P.); chensq@ysfri.ac.cn (C.S.); liangzr@ysfri.ac.cn (L.Z.); wangwj@ysfri.ac.cn (W.W.); sunxt@ysfri.ac.cn (S.X.); 2 Laboratory for Marine Fisheries Science and Food Production Processes, Qingdao National Laboratory for Marine Science and Technology, Qingdao 266071, China

**Keywords:** *Saccharina japonica*, ubiquitination, heat stress

## Abstract

Ubiquitination is a major post-translational modification involved in nearly all aspects of eukaryotic biology. Previous RNA-Seq studies showed that ubiquitination plays essential roles in the heat tolerance of *Saccharina japonica*, but to date, large-scale profiling of the ubiquitome in *S. japonica* has not been reported. To better understand the regulatory roles of ubiquitination in heat responses of *S. japonica*, we investigated its ubiquitome under normal and heat stress by the combination of affinity enrichment and high-resolution liquid chromatography-tandem mass spectroscopy analysis. Altogether, 3305 lysine ubiquitination sites in 1562 protein groups were identified. After normalization, 152 lysine ubiquitination sites in 106 proteins were significantly upregulated and 208 lysine ubiquitination sites in 131 proteins were significantly downregulated in response to heat stress. Protein annotation and functional analysis suggested that ubiquitination modulates a variety of essential cellular and physiological processes, including but not limited to the ubiquitin-26S proteasome system, ribosome, carbohydrate metabolism, and oxidative phosphorylation. Our results provide a global view of the heat response ubiquitome in *S. japonica*, and could facilitate future studies on the physiological roles of these ubiquitination-related proteins.

## 1. Introduction

*Saccharina japonica* is one of the most important aquacultured brown algae with world’s annual yield of eleven million tons, and has significant economic, social, and ecological values (FAO, http://www.fao.org/). *S. japonica* is a kind of cold-temper kelp, native to the waters near Hokkaido, Japan. However, the current major aquaculture areas of this kelp are distributed in the temperate zone (Shandong and Liaoning Provinces of China) or subtropical zone (Fujian Province, China). Comparing with the origin place, the water temperature of cultivation zone is higher, so the kelp is always subjected to heat stress [1]. In addition, as global temperatures get higher, the growth period of *S. japonica* is becoming shorter and the disease risk during the seedling breeding and cultivation increases, which will limit the further improvement of the yield and quality of this kelp [2]. It is significant to breed heat resistance varieties for the healthy and sustainable development of *S. japonica* aquaculture. In comparison with traditional cross-breeding, molecular breeding has its advantages in high efficiency and short period. Hence, it is necessary to elucidate the heat responsive mechanism and identify more functional genes which are involved in the heat tolerance in *S. japonica*.

Similar to high order plants, algae have developed sophisticated mechanisms to tolerate, acclimate, and survive adverse conditions. Several studies have paid attention to the physiological and biochemical responses of *S. japonica* to heat stress, and demonstrated that high temperature could trigger the decrease of growth rate and photosynthetic efficiency, and increase the activity of antioxidative enzymes in *S. japonica* [1,3]. To further explore the regulatory mechanisms, Liu et al. investigated the heat stress response of *S. japonica* at both transcriptome and proteome levels, identifying hundreds of the differentially expressed genes/proteins and miRNAs involved in “protein processing in endoplasmic reticulum”, “metabolic pathway”, “biosynthesis of secondary metabolites”, and “protein and amino acid metabolism”, etc. [2,4,5]. Notably, these omics data showed that under heat stress the ubiquitination-related proteins changed significantly at both transcription and translation levels, and several differential miRNAs also targeted ubiquitin ligases, indicating that ubiquitination may play important roles in the heat tolerance of *S. japonica*. 

Ubiquitination is a specific ubiquitin (a small globulin molecule composed of 76 amino acids) modification process of target proteins [6]. This process occurs through a sophisticated three-step enzymatic cascade involving ubiquitin-activating enzymes (E1) [7], ubiquitin-conjugating enzymes (E2) [8], and ubiquitin ligases (E3) [9,10]. During this process, E1 catalyzes the ATP-dependent activation of ubiquitin and formation of a thioester bond between ubiquitin and catalytic cysteine on the E1. Ubiquitin is then transferred to a catalytic cysteine of E2 and through the E3 to the substrate. Ubiquitination typically leads to the formation of an amide linkage comprising the ε-amine of lysine (Lys) of the target protein and the C terminus of ubiquitin [11]. Based on the bonding form, the ubiquitination modification of proteins is classified into monoubiquitylation, multiubiquitylation, and polyubiquitylation [12]. The mono- or multi-ubiquitylation has been shown to be required for recruiting binding partners, inhibiting interactions, changing protein localizations, or modulating protein activities [8,13,14]. Whereas, the most famous fate of polyubiquitinated proteins is their degradation by the 26S proteasome, named ubiquitin-26S proteasome system (UPS), thus playing key roles in the plant biology, including growth and development, abiotic or biotic stresses, immunity, and hormonal signaling [15,16,17]. Except that, polyubiquitylation is also involved in DNA damage repair, regulation of transcription, translation, and endocytic trafficking, etc. [13,16,18].

Many studies have shown that ubiquitination plays key roles in the heat response of plant [19,20]. Ubiquitination modification can promote the stress signals transduction through positive regulation [21] or act as a negative regulator to degrade target proteins via UPS [22,23,24]. To that in algae, several studies pointed out that ubiquitination-related proteins were significantly regulated under heat stress [25,26,27,28,29]. To date, no large-scale ubiquitome profiling under heat stress of algae has been reported. 

In this study, we firstly report the ubiquitome profiling in *S. japonica*, and investigated the potential changes of ubiquitinated proteins under heat stress via affinity enrichment and LC-MS/MS approach. This study provides a global view of the function of ubiquitination in heat response of *S. japonica*, paving the ways for illustrating the ubiquitin-driven regulatory mechanism and shedding light on the improvement of heat tolerance in *S. japonica*.

## 2. Results

### 2.1. Overview of the Ubiquitomic Analysis

**Figure 1:**
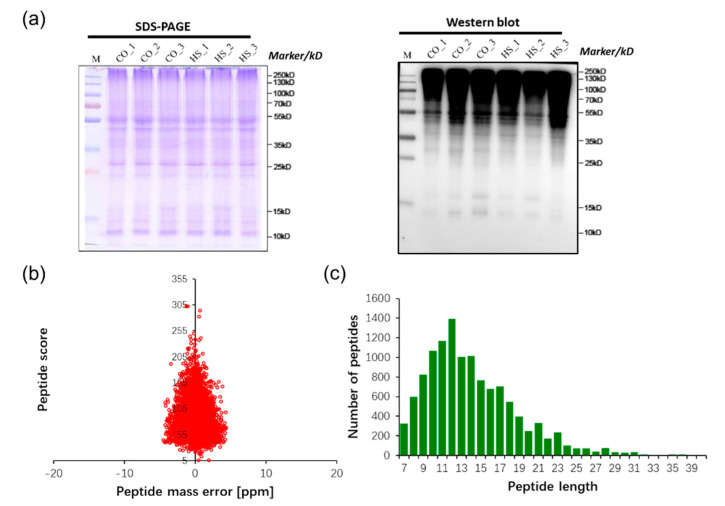
Proteomic identification of ubiquitinated peptides in *Saccharina japonica*. (**a**) Sodium dodecyl sulfate polyacrylamide gel electrophoresis (SDS-PAGE) and Western blot analysis using an anti-ubiquitin antibody. (**b**) Mass error distribution of all identified peptides by normal and heat stress. (**c**) Peptide length distribution.

To test the relative abundance of ubiquitinated proteins under normal temperature and heat stress, proteins extracted from normal temperature and heat stress treatments were subjected to Western blot analysis with an anti-ubiquitin antibody. It was obvious that proteins with a wide range of molecular masses were ubiquitinated, and it is worth noting that a slight immunoblot signal disappeared between 25 and 35 kD after heat stress treatments (Figure 1a). To further identify the different ubiquitinated proteins, the lysine-ubiquitinated peptides were enriched using anti-KGG antibody beads and were detected using LC-MS/MS. The distribution of mass error of all the identified peptides was near zero and most of them were less than 0.02 Da, meaning the mass accuracy of the MS data fit the requirement (Figure 1b). The length of most peptides distributed between 7 and 40, meaning that sample preparation reached the standard (Figure 1c).

### 2.2. Properties of Ubiquitination Sites and Motifs

We evaluated the number of ubiquitinated sites in the identified proteins. Altogether, 3305 lysine ubiquitination sites in 1562 proteins were identified, among which 1620 lysine ubiquitination sites in 917 proteins were quantified, and 634 lysine ubiquitination sites in 264 proteins were normalized. In this study, the quantitative ratio over 1.5 was considered upregulation while quantitative ratio below 0.67 was considered as downregulation. Based on this criterion, 152 lysine ubiquitination sites in 106 proteins were upregulated and 208 lysine ubiquitination sites in 131 proteins were downregulated in response to heat stress (Table 1 and Table S1).

**Figure 2:**
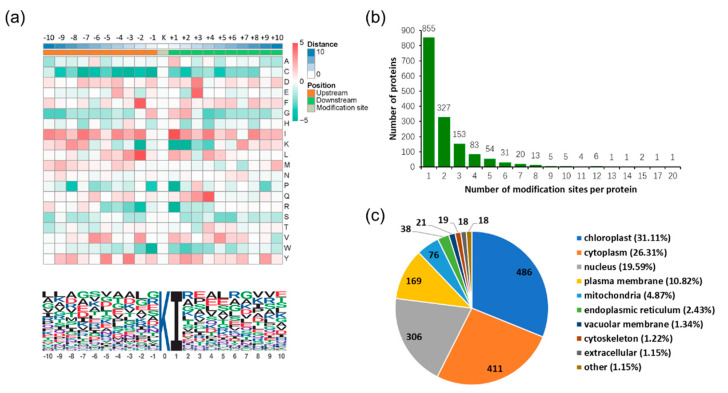
Ubiquitinated lysine motifs and proteins in *S. japonica*. (**a**) Motif analysis of all the identified sites. In the heat map, the *p* value matrix was transformed by the function x = log10 (*p* value) when fold change <1, and transformed by the function x = −log10 (*p* value) when fold change >1. The red indicates that this amino acid is significantly enriched, and green indicates that this amino acid is significantly reduced. The height of each letter in the conserved domain corresponding to the frequency of the amino acid residue in that position. (**b**) The number of modified sites in the detected diglycine-modified peptides. (**c**) The subcellular location of all differential proteins.

To further understand the ubiquitinated sites structures in *S. japonica*, we summarized the common amino acid sequences around the modified lysine residue (Figure 2a). The results showed that 171 peptides featured an isoleucine at the +1 position, the other positions in the range of −10 to +10 position exhibited considerable sequence variability (Figure 2a). Among the ubiquitinated proteins, 54.74% of which had only one ubiquitinated lysine site, and 43.9% proteins had 2–9 ubiquitinated sites. In addition, 17 proteins (1.09%) were ubiquitinated at 10 or more lysine sites (Figure 2b). Furthermore, according to subcellular location annotation information of identified proteins, the amount of the identified proteins in each subcellular location were added up. The results showed that 486 proteins were located in the chloroplast, accounting for 31.11% of all the proteins, and other 411, 306, 169, 76, 38, 21, 19, 18, and 18 proteins were located in cytoplasm, nucleus, plasma membrane, mitochondria, endoplasmic reticulum, cytoskeleton, vacuolar membrane, cytoskeleton, and extracellular, accounting for 26.31%, 19.59%, 10.82%, 4.87%, and 2.43%, 1.34%, 1.22%, 1.15%, and 1.15%, respectively (Figure 2c).

### 2.3. Gene Ontology (GO) Functional Classification and GO Enrichment-Based Clustering Analysis of the Differentially Expressed Ubiquitinated Proteins

**Figure 3:**
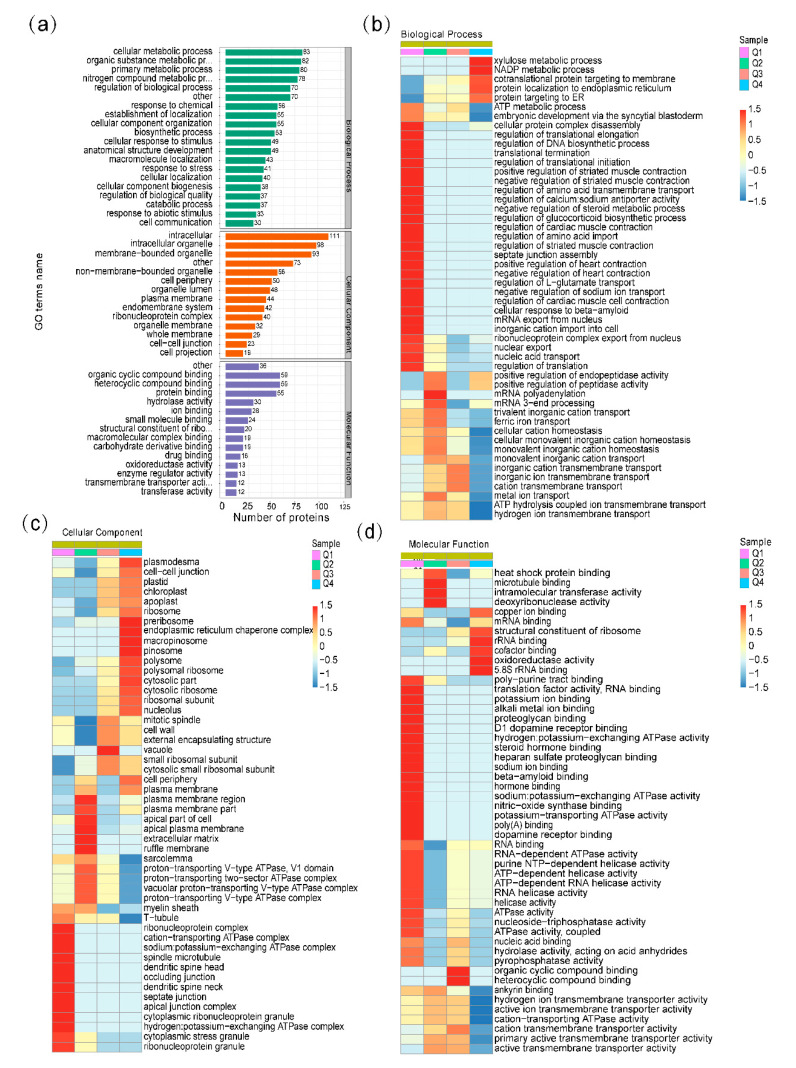
GO functional classification and GO enrichment-based clustering analysis. (**a**) GO functional classification of the differential ubiquitinated proteins based on gene ontology; (**b**–**d**) GO enrichment-based clustering analysis of the differentially ubiquitinated proteins. Those categories at least enriched in one of the clusters with *p* value < 0.05 were clustered. The *p* value matrix was transformed by the function x = −log10 (*p* value), and was then z-transformed for each category. These z scores were clustered by one-way hierarchical clustering and visualized by a heat map using R-package. (**b**) Biological process analysis; (**c**) cellular component analysis; (**d**) molecular function analysis.

To understand the function of ubiquitination in response to the heat stress of *S. japonica*, Gene Ontology (GO) functional classification of the differential ubiquitinated proteins was conducted based on the biological processes, molecular functions, and cellular components (Figure 3). In the biological process category, proteins were relatively enriched in the cellular metabolic process (8%), organic substance metabolic process (8%), primary metabolic process (7%), nitrogen compound metabolic process (7%), and regulation of biological process (6%) (Figure 3a). The three principal cellular components were intracellular (15%), intracellular organelle (13%), and membrane-bounded organelle (12%) (Figure 3a). For the molecular function analysis, we found that the proteins related to organic cyclic compound binding (14%), heterocyclic compound binding (14%), and protein binding (13%) were enriched (Figure 3a).

To better reveal the changes of ubiquitinated protein in response to heat stress, GO enrichment-based clustering analyses were performed by dividing all the differentially expressed ubiquitinated proteins into four quantiles: Q1 (quantitative ratio 0–0.5), Q2 (quantitative ratio 0.5–0.67), Q3 (quantitative ratio 1.5–2), and Q4 (quantitative ratio >2) (Figure 3b–d, Table S2). In the biological process category, xylulose metabolic process, NADP metabolic process, cotranslational protein targeting to membrane, protein localization to endoplasmic reticulum, and protein targeting to ER were found to be significantly enriched in Q4 with upregulated lysine ubiquitination. While, the cellular protein complex disassembly, regulation of DNA and protein biosynthetic process, and regulation of molecular transport were enriched in Q1 with downregulated lysine ubiquitination (Figure 3b). In the cellular component category, the upregulated proteins were highly enriched in plasmodesma, preribosome, endoplasmic reticulum chaperone complex, macropinosome, pinosome, and vacuole, while the downregulated proteins were enriched in cell membrane, extracellular matrix, ribonucleoprotein complex, ATPase complex, and cytoplasmic stress granule (Figure 3c). In the molecular function category, proteins involved in structural constituent of ribosome, rRNA binding, cofactor binding, oxidoreductase activity, 5.8S rRNA binding, organic cyclic compound binding, and heterocyclic compound binding were upregulated and enriched in Q3 and Q4, while proteins involved in heat shock protein binding, deoxyribonuclease activity, translation factor activity, ion binding, proteoglycan binding, ATPase activity, hormone binding, and nitric-oxide synthase binding were downregulated and enriched in Q1 and Q2 (Figure 3d).

### 2.4. Kyoto Encyclopedia of Genes and Genomes (KEGG) Pathway Analysis of the Differentially Expressed Ubiquitinated Proteins

**Figure 4:**
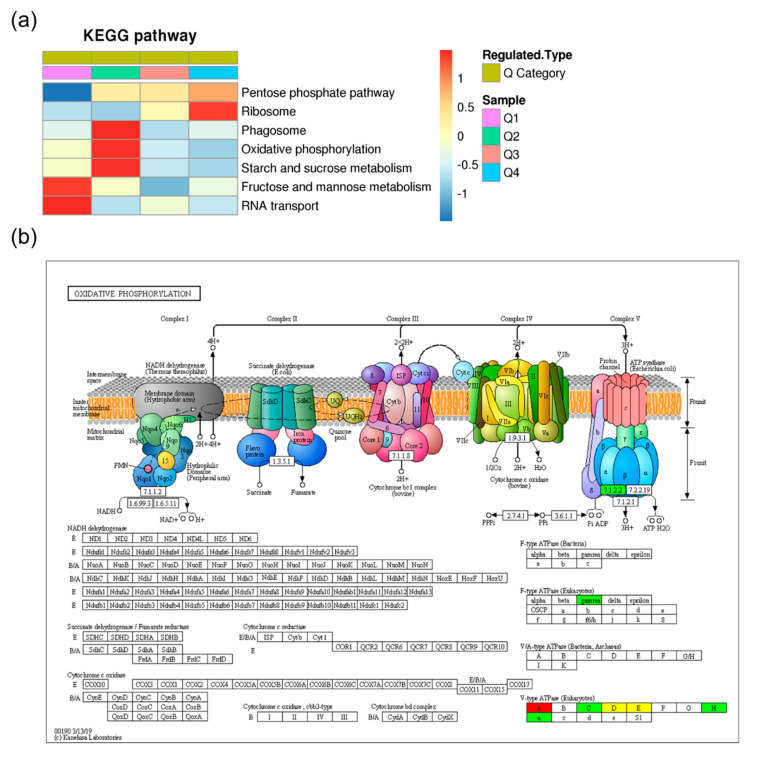
KEGG pathway analysis of the differentially expressed lysine ubiquitinated proteins. (**a**) KEGG pathway-based clustering analysis of the differentially expressed ubiquitinated proteins. Those pathways at least enriched in one of the clusters with *p* value < 0.05 were clustered. The *p* value matrix was transformed by the function x = −log10 (*p* value), and were then z-transformed for each category. These z scores were clustered by one-way hierarchical clustering and visualized by a heat map using R-package; (**b**) the pathway obtained from KEGG pathway enrichment analysis. The colors reflect ubiquitinated level upregulated (red), downregulated (green), or both upregulated and downregulated at different modification sites (yellow).

To identify pathways which were differentially ubiquitinated under heat stress, the Kyoto Encyclopedia of Genes and Genomes (KEGG) pathway-based clustering analysis was conducted. The results showed that the pathways of phagosome, oxidative phosphorylation, starch and sucrose metabolism, fructose and mannose metabolism, and RNA transport were enriched in the downregulated ubiquitinated proteins, while the upregulated ubiquitinated proteins were only significantly enriched in the ribosome pathway (Figure 4a, Table S3). Changes in one of the most significantly enriched pathways, oxidative phosphorylation, are shown in Figure 4b, which indicated that both the V-type ATPase and F-type ATPase showed differential ubiquitination. 

### 2.5. Protein Domain Based Enrichment Analysis of the Differentially Expressed Lysine Ubiquitinated Proteins

**Figure 5:**
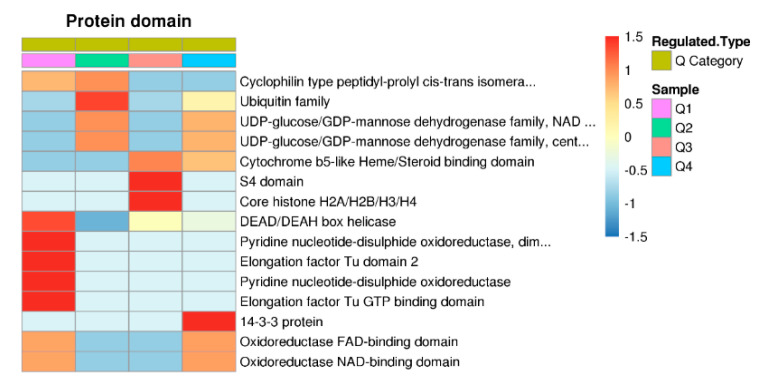
Protein domain based clustering analysis. Those domains at least enriched in one of the clusters with *p* value < 0.05 were clustered. The *p* value matrix was transformed by the function x = −log10 (*p* value), and was then z-transformed for each domain. These z scores were clustered by one-way hierarchical clustering and visualized by a heat map using R-package.

To address the domain features of the ubiquitinated proteins, the protein domain annotation and clustering analysis were performed (Table S4). The results showed that ubiquitin family domain, DEAD/DEAH box helicase, pyridine nucleotide−disulphide oxidoreductase domains, and elongation factor Tu domains were downregulated and highly enriched in Q1 or Q2 (Figure 5). In the upregulated proteins, various domains, such as cytochrome b5-like Heme/Steroid binding domain, S4 domain, core histone H2A/H2B/H3/H4, and 14-3-3 protein domains were highly enriched. In addition, UDP-glucose/GDP-mannose dehydrogenase family domains and oxidoreductase domains were enriched in the upregulated category (Q3/Q4) and the downregulated category (Q1/Q2) at the same time (Figure 5).

### 2.6. Protein–Protein Interaction (PPI) Network Analysis of Differentially Expressed Ubiquitinated Proteins

**Figure 6:**
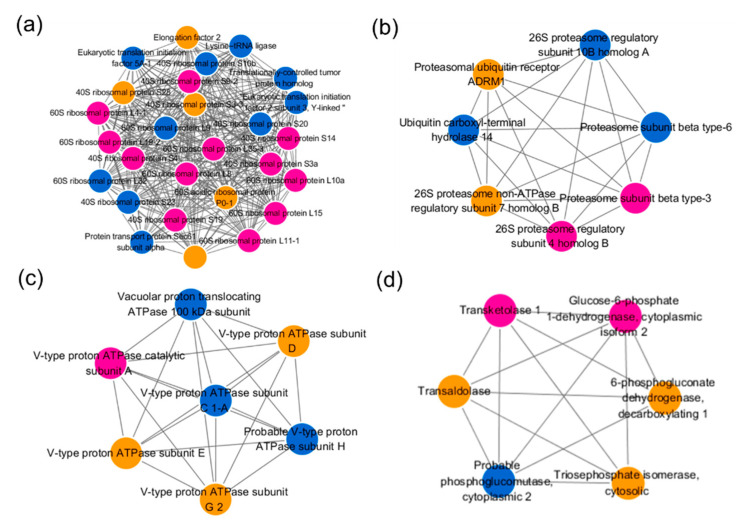
Interaction network of the quantified lysine ubiquitinated proteins. (**a**) The subnetwork involved in ribosome pathway; (**b**) the subnetwork involved in UPS; (**c**) the subnetwork related with V-type ATPase; (**d**) the subnetwork related with pentose phosphate pathway. The proteins in purple were upregulated and the proteins in blue were downregulated. Proteins in orange were modified at different sites with both increased and decreased levels.

To identify the key nodes and important connectors among the differentially expressed ubiquitinated proteins under heat treatment, the protein–protein interaction network analysis was performed, and the subnetwork was filtered by mcode function in cytoscape (Table S5). As shown in Figure 6, the most abundant subnetwork is involved in ribosome pathway which contained 27 ubiquitinated proteins. Among which, the ubiquitinated levels of 12 proteins were upregulated, while 10 proteins were downregulated, and the remaining five proteins contained both upregulated and downregulated sites (Figure 6a). As expected, seven ubiquitinated proteins related with UPS were identified and all of them interacted with each other (Figure 6b). Similarly, the subnetwork containing seven ubiquitinated proteins related with V-type ATPase was built and the involved proteins interacted with each other (Figure 6c). In addition, six enzymes involved in pentose phosphate pathway were filtered and formed a subnetwork (Figure 6d).

## 3. Discussion

### 3.1. Ubiquitination Involved in the Heat Response of S. japonica

Heat is one of the most common environmental stressors widely threatening the growth, development, and yield of plants and algae. As a well conserved post-translational modification, ubiquitination has been shown to play crucial roles in the heat tolerance of eukaryotes, but the corresponding regulatory mechanism is still far from interpretation [5,20,26,27]. To comprehensively identify the differential ubiquitinated proteins, we firstly conducted a comparative proteomics study of lysine ubiquitination in *S. japonica* by the combination of affinity enrichment and LC-MS/MS analysis. Totally, we identified 3305 lysine ubiquitination sites in 1562 proteins in *S. japonica*. Using the similar method, 861 di-Gly-Lys-containing peptides in 464 proteins in rice leaf, 433 lysine ubiquitination sites in 285 proteins in wheat seedlings, and 544 lysine ubiquitination sites in 352 proteins in peach leaves have been identified [15,30,31]. In addition, Walton et al. identified 3009 sites on 1607 proteins in *Arabidopsis thaliana* by ubiquitin combined fractional diagonal chromatography (COFRADIC) method [32]. A comparison of our results to those studies suggested that the reported ubiquitome size of the *S. japonica* is similar to that in *A. thaliana*, and higher than that in the leaves or seedlings of wheat, rice, and peach. 

Although the ubiquitome have been reported in several plant species, our understanding on global changes in the ubiquitome under heat response is still lacking. Previous studies have revealed that heat treatment caused increase of ubiquitinated proteins with high molecular weight (MW) in *Chlamydomonas reinhardii* cells and wheat roots, and decrease of ubiquitinated proteins with MW around 30 kDa [33,34,35]. In this study, we compared the ubiquitinated proteins obtained from samples untreated/treated with high temperature. Although it is not clear, it could be found that an ubiquitinated proteins band with MW ≈ 30 kDa disappeared, which was in accordance with previous studies, while we were unable to find an increase of high molecular mass associated with heat stress which was consistent with the result from pear cells [36].

### 3.2. The UPS-Related Protein Showed Differential Ubiquitination

Ubiquitination starts by the covalent attachment of one or more ubiquitin (Ub) to a Lys (K) residue within specific target proteins [20]. Since Ub contains seven Lys residues (Lys6, Lys11, Lys27, Lys29, Lys33, Lys48, and Lys63) itself, Ub molecules can form different types of polyubiquitin [37]. In this study, four ubiquitination sites in Ub (Novel01052) were identified as differential ubiquitinated sites, among which, Lys6, Lys11, and Lys63 had increased ubiquitination levels while Lys48 had decreased ubiquitination level (Table S1). Given that Lys48- and Lys11-linked ubiquitin chains target proteins for selective degradation by the UPS [8,38], while Lys63-linked ubiquitin chains are involved in both proteasome-independent cellular processes such as DNA repair, signal transduction, and receptor endocytosis and serving as targeting signals for UPS [13,17], we supposed that UPS is involved in the heat responses of *S. japonica*. Except for the ubiquitin protein, the UPS also includes 26S proteasome, the ubiquitin conjugation cascade (E1, E2, E3), and deubiquitinating enzymes [39]. In the 26S proteasome, two core subunits (proteasome subunit beta type-3, proteasome subunit beta type-6) and three regulatory particles (26S proteasome regulatory subunit 4, 26S proteasome regulatory subunit 10B, and 26S proteasome non-ATPase regulatory subunit 7) were found to be differential ubiquitinated (Figure 6b). Additionally, other UPS components including the ubiquitin-like modifier-activating enzyme 1 (JXRI01000719.1.2) showed an increase ubiquitinated level, while ubiquitin ligase complex E3 (JXRI01000954.1.4), ubiquitin carboxyl-terminal hydrolase 14 (JXRI01000061.1), and proteasomal ubiquitin receptor ADRM1 (JXRI01001636.1) showed decreased levels. The ubiquitin-mediated degradation is employed to remove misfolded and misassembled proteins which synthesized at the endoplasmic reticulum (ER), named ER-associated degradation (ERAD) [40]. Numerous studies had described that the molecular chaperones augment protein biogenesis in the ER and mediate ERAD substrate selection [41]. We observed that the ubiquitination levels of six molecular chaperones, including one HSP90 (JXRI01001002.1.7), three HSP70s (JXRI01000196.1.11, JXRI01000260.1.2, and JXRI01000101.1.18), one Hsp70-Hsp90 organizing protein 3 (JXRI01000156.1.12.6), and one HSP40 (JXRI01000404.1.4) were significantly regulated, which further proved the regulatory effect of UPS in the heat response of kelp.

### 3.3. Key Biological Processes are Modified by Ubiquitination under Heat Stress

Further enrichment analysis in our current study suggested that ubiquitination plays a key role in regulating diverse biological processes. As a factory for protein synthesis in cells, the ribosome is an extremely crucial structure in the cell. It has been proved that multiple ribosomal subunits were abundantly ubiquitinated in *Arabidopsis*, wheat, peach, and human [15,31,42,43]. In present study, at least 27 ubiquitylated related proteins of the 40S and 60S ribosome complexes in *S. japonica* were significantly upregulated or downregulated (Figure 6a; Table S1), implying that ubiquitination of ribosomal proteins is likely to be an important regulatory mechanism in heat response of *S. japonica*. Previous studies showed that the ubiquitination of mature ribosomes function in the selective degradation by autophagy upon nutrient starvation in *Saccharomyces cerevisiae* [44], and are a process of the ribosome quality control machinery [45,46]. Thus, we speculated that heat stress leads to the stalling, collisions, improper folding, and non-function of ribosomes that trigger a series of quality control events to maintain protein homeostasis and cellular fitness, such as selective degradation by autophagy and UPS [46,47].

The eukaryotic V-type adenosine triphosphatase (V-ATPase) is a multi-subunit membrane protein complex that functions as an ATP-dependent proton pump [48], and functions in processes such as receptor-mediated endocytosis, intracellular targeting of lysosomal enzymes, protein processing, and degradation [49]. The V-ATPase is composed of two distinct protein complexes, V_0_ and V_1_ [50]. Seol et al. have reported that Skp1–Cul1–F-box (SCF) ubiquitin ligases are associated with the V_1_ domain of the V-ATPase, and promote glucose-triggered assembly of the V-ATPase holoenzyme [51]. Among those subunits, six V_1_ subunits had significantly differential ubiquitinated levels (Figure 4b and Figure 6c). The ubiquitinated level of V-ATPase catalytic subunit A (JXRI01000157.1.11) was upregulated, and the ubiquitinated level of subunit H (JXRI01000205.1) and subunit C (JXRI01000107.1.43) were downregulated, while the ubiquitinated level of subunit E (JXRI01000452.1), subunit D (JXRI01001485.1.7), and subunit G 2 (JXRI01000315.1) were upregulated/downregulated at different sites. Additionally, the V_0_ subunit Vacuolar proton translocating ATPase 100 kDa subunit (JXRI01001466.1) had a significantly decreased ubiquitinated level. Our data imply that the ubiquitination of V-ATPase is likely to be a crucial regulatory mechanism in the heat response of *S. japonica*. What is more, the ubiquitinated level of a F-type ATPase gramma subunit (JXRI01000589.1.3.1) is also downregulated, hinting its regulation function in the heat stress response.

Diverse carbohydrate metabolisms were found to be differential ubiquitylation significantly under high temperature. Notable examples include those proteins involved in the pentose phosphate pathway (PPP) (Figure 6d, Table S1). The PPP could produce ribose 5-phosphate and nicotinamide adenine dinucleotide phosphate (NADPH) for the biological synthesis, and contribute to the growth and development and the abiotic stresses responses of plants [52,53]. Glucose-6-phosphate dehydrogenase (G6PDH, JXRI01000033.1.28) and 6-phosphogluconate dehydrogenase (6PGDH, JXRI01000033.1.27) are two key enzymes in the PPP, the ubiquitination level of all ubiquitinated sites of G6PDH and five ubiquitinated sites of 6PGDH were found upregulated. In addition, PPP related transketolase 1 (JXRI01000333.1.14, Up), phosphoglucomutase (JXRI01000641.1.7, Down), transaldolase (JXRI01000034.1.3, Up and down) were also differentially ubiquitinated under heat stress. The alginates, fucoidans and cellulose constitute the cell wall of mature intertidal brown algae, and take part in the heat stress response of the kelp [15,54,55,56]. As the rate-limiting enzyme in the alginate biosynthetic pathway, the ubiquitinated level of two GDP-mannose dehydrogenases (JXRI01001435.1.1 and JXRI01000030.1.16) was significantly regulated. Our ubiquitome data also indicated that phosphomannomutase (JXRI01000044.1.5) which is involved in the biosynthetic pathway of alginate and fucoidan, was clearly decreased in ubiquitination under heat condition, and the GDP-mannose 4,6-dehydratase 1 (JXRI01000145.1.24) and GDP-fucose pyrophosphorylase (JXRI01000175.1.10) in the biosynthetic pathway of fucoidan also showed a significant up/downregulated ubiquitinated level. In addition, the ubiquitinated level of cellulose synthase (JXRI01001136.1.7) in the biosynthetic pathway of cellulose was also significantly regulated. These findings indicated that the kelp might survive in heat condition by regulating the synthesis of polysaccharides [57]. Additionally, our experimental data indicated that other carbohydrate metabolisms, such as mannose metabolism, glycolysis/gluconeogenesis, and tricarboxylic acid (TCA) cycle are also significantly regulated by ubiquitin modification.

Overall, this study provides the first extensive data on lysine ubiquitination in kelp seaweeds, and demonstrated that ubiquitination plays a widespread role in the heat response of *S. japonica* by modulating diverse cellular processes. Given the diversity of ubiquitination modification, the function and regulatory mechanism of the key targets and key pathways may be well worth further validating and exploring. Our findings lay a foundation for further functional analysis of lysine ubiquitination in the heat response of *S. japonica*.

## 4. Materials and Methods

### 4.1. Sample Preparation

*S. japonica* juvenile sporophytes (Huangguan No.1) with the length of 10–15 cm were collected from cultivated rafts in Rongcheng, China. The samples were washed with sterile seawater and precultured in filtered sterilized seawater at 10 °C under a 10 h/14 h light/dark photoperiod with 35 ± 5 μmol photons m^−2^ s^−1^ light intensity. Then, 10 healthy individuals were taken as the control group (marked as CO) and still cultured at 10 °C under 35 ± 5 μmol photons m^−2^ s^−1^ light intensity for 3 h, and the other 10 individuals were taken as the treatment group (marked as HS) and cultured at 20 °C under 35 ± 5 μmol photons m^−2^ s^−1^ light intensity for 3 h. All individuals in CO and HS groups were mixed separately and were frozen in liquid nitrogen, followed by storing at −80 °C until protein extraction.

### 4.2. Protein Extraction

Samples were grinded with liquid nitrogen, then the cell powder was sonicated three times on ice in lysis buffer (8 M Urea, 10 mM Dithiothreitol (DTT), 50 mM Nicotinamide (NAM), 3 µM Trichostatin A (TSA), and 0.1% Protease Inhibitor Cocktail) using a high intensity ultrasonic processor. Following lysis, the suspension was centrifuged at 20,000 g for 20 min at 4 °C to remove the debris. Then the protein was precipitated with ice-cold 15% trichloroacetic acid (TCA) at −20 °C for 2 h. The mixture was centrifuged at 4 °C for 10 min. The supernatant was discarded, and the pellet was washed with cold acetone three times. Finally, the protein was resuspended in buffer (8 M urea, 100 mM NH_4_CO_3_, pH 8.0) and the protein concentration was quantified with 2-D Quant kit according to the manufacturer’s instructions.

### 4.3. Trypsin Digestion

A total of 100 μg protein sample was treated with 5 mM DTT at 56 °C for 30 min and alkylated with 11 mM iodoacetamide (IAA) at room temperature for 15 min in darkness. Then the sample was diluted by adding 200 mM triethylammonium bicarbonate (TEAB) to urea concentration less than 2 M. After that, they were digested with trypsin at a trypsin-to-protein mass ratio of 1:50 for the first digestion overnight and a trypsin-to-protein mass ratio of 1:100 for a second digestion for 4 h.

### 4.4. High Performance Liquid Chromatography (HPLC) Fractionation

The sample was then fractionated into fractions by high pH reverse-phase HPLC using Agilent 300 Extend C18 column (5 μm particles, 4.6 mm inner diameter, 250 mm length). Briefly, peptides were separated using a gradient of increasing acetonitrile concentration (2–60% linear gradient in 80 min) into 80 fractions. Then the peptides were combined into four fractions for each sample and dried by vacuum centrifuging.

### 4.5. Affinity Enrichment of Ubiquitinated Peptides

The tryptic peptides were dissolved in NETN buffer consisting of 100 mM NaCl, 1 mM Ethylene diamine tetraacetic acid (EDTA), 50 mM Tris-HCl, and 0.5% Nonidet P (NP-40) at pH 8.0. The solution was incubated with prewashed K-ε-GG antibody beads (PTM Biolabs, Hangzhou, China), and gently shaken at 4 °C overnight. The beads were washed with NETN buffer four times and with ddH_2_O twice. The di-Gly-Lys-containing peptides were eluted from the beads with 0.1% trifluoroacetic acid (TFA). Finally, the eluted fractions were combined and vacuum-dried, and the resulting peptides were cleaned with C18 ZipTips (Millipore, Billerica MA, USA).

### 4.6. Liquid Chromatography (LC)-Tandem Mass Spectroscopy (MS/MS) Analysis 

The peptides were dissolved in 0.1% formic acid (FA), directly loaded onto a reversed-phase pre-column (Acclaim PepMap 100, Thermo, Waltham, MA, USA). Peptide separation was performed using a reversed-phase analytical column (Acclaim PepMap RSLC, Thermo). The gradient was composed of an increase from 7% to 25% solvent B (0.1% FA in 98% acetonitrile) for 40 min, 25% to 40% for 12 min, and climbing to 80% in 4 min then holding at 80% for the last 4 min, all at a constant flow rate of 400 nL/min on an EASY-nLC 1000 ultra performance liquid chromatography (UPLC) system (Thermo, Waltham, MA, USA).

The enriched ubiquitinated peptides were subjected to nanospray ionization (NSI) source and MS/MS in Q Exactive Plus^TM^ (Thermo) coupled online to the UPLC. Intact peptides were detected in the orbitrap with mass resolution of 70,000. Peptides were selected for MS/MS using normalized collision energy (NCE) setting of 28, and ion fragments were detected in the orbitrap with mass resolution of 17,500. For the top 20 precursor ions above a threshold ion count of 5E3, a data-dependent procedure that alternated between one MS scan followed by 20 MS/MS scans with 15.0 s dynamic exclusion were applied. The electrospray voltage applied was 2.0 kV. Automatic gain control (AGC) was 5E4. The m/z scan range was set as 350–1800. Fixed first mass was 100 m/z.

### 4.7. Database Search and Quantification

The MS/MS data was processed using MaxQuant (http://www.maxquant.org/) with integrated Andromeda search engine (v.1.5.2.8, Martinsried, Germany). Tandem mass spectra were searched against *S. japonica* genome database (GenBank: GCA_000978595.1) [54] concatenated with reverse decoy database. Trypsin/P was specified as cleavage enzyme allowing up to two missing cleavages, five modifications per peptide, and five charges. Minimum peptide length was set at 7. The first order parent ion mass error was set to 0.07 and 0.006 Da in first search and main search, and the second order parent ion mass error was set to 40 ppm [58]. Carbamidomethylation on Cys was specified as fixed modification and oxidation on Met, GlyGly on Lys, and Acetylation on protein N-terminal were specified as variable modifications. False discovery rate (FDR) thresholds were specified at 1%. The site localization probability was set as ≥0.75. The relative quantification between two samples is the quantification values ratio of the two samples. To remove the effect of protein expression on modification abundance, normalization with protein quantification [5] was used for subsequent bioinformatics analysis. 

### 4.8. Bioinformatics Methods

The protein domains were annotated by InterProScan (http://www.ebi.ac.uk/interpro/) using InterPro domain database based on protein sequence alignment method. MoMo Soft (motif-x algorithm, http://meme-suite.org/tools/momo) was used to analyze the model of sequences constituted with amino acids in specific positions of modify-21-mers (10 amino acids upstream and downstream of the site) in all protein sequences. The wolfpsort soft (http://www.genscript.com/psort/wolf_psort.html) was used to predict subcellular localization. Gene Ontology (GO) annotation proteome was derived from the UniProt-GOA database (http://www.ebi.ac.uk/GOA/). Kyoto Encyclopedia of Genes and Genomes (KEGG) database online service tools KAAS was used to annotate the protein pathway. A two-tailed Fisher’s exact test was employed to test the enrichment of the different protein functional classifications (Domain, GO, Pathway), against all database proteins. The quantified proteins in this study were divided into four quantiles according to the quantification ratio to generated four quantiles: Q1 (0–0.5), Q2 (0.5–0.67), Q3 (1.5–2), and Q4 (>2). Then, the quantiles-based clustering was performed. Cluster membership was visualized by a heat map using the “heatmap.2” function from the “gplots” R-package. Correction for multiple hypothesis testing was carried out using standard false discovery rate control methods and functional classification with a corrected *p*-value < 0.05 was considered significant. The candidate differentially modified proteins database accession or sequence were searched against the STRING database version 11.0 (https://string-db.org/) for protein–protein interactions. Interaction network form STRING was visualized in Cytoscape 3.3.0 (Cambridge, MA, USA) [59].

### 4.9. Western Blot Analysis

To examine ubiquitination levels, total proteins were extracted from *S. japonica* juvenile sporophytes that were collected at 0 and 3 h under heat stress. The proteins were analyzed by 10% sodium dodecyl sulfate polyacrylamide gel electrophoresis (SDS-PAGE). Ubiquitination levels were then determined by immunoblotting with primary anti-ubiquitin antibody (1:2000, PTM BIO), followed by secondary anti-rabbit antibody conjugated to horseradish peroxidase (HRP) (1:10,000, PTM BIO). Finally, the chemiluminescence signal was visualized with an X-ray film.

## Figures and Tables

**Table 1:** Summary of differentially quantified sites and proteins after normalization.

Name	Upregulated (>1.5)	Downregulated (<0.67)
Sites	152	208
Proteins	106	131

## Supplementary Materials

Supplementary Materials can be found at https://www.mdpi.com/1422-0067/21/21/8210/s1. Table S1. Differentially expressed statistics; Table S2. GO_enrichment data; Table S3. KEGG_pathway_enrichment data; Table S4. Protein domain enrichment data; Table S5. Network statistics.
